# Theoretical investigation of the ORR on boron–silicon nanotubes (B–SiNTs) as acceptable catalysts in fuel cells

**DOI:** 10.1039/c9ra05031k

**Published:** 2019-10-04

**Authors:** Razieh Razavi, Meysam Najafi

**Affiliations:** Department of Chemistry, Faculty of Science, University of Jiroft Jiroft Iran R.Razavi@ujiroft.ac.ir; Medical Biology Research Center, Health Technology Institute, Kermanshah University of Medical Sciences Kermanshah Iran iau.mnajafi@yahoo.com

## Abstract

Here, the potential of boron doped silicon nanotubes (7, 0) as ORR catalysts is examined. Acceptable paths for the ORR on studied catalysts are examined through DFT. The optimum mechanism of the ORR on the surface of B_2_–SiNT (7, 0) is shown. The ORR on the surface of B_2_–SiNTs (7, 0) can continue through LH and ER mechanisms. The calculated beginning voltage for the ORR on B_2_–SiNTs (7, 0) is 0.37 V and it is smaller than the beginning voltage (0.45 V) for platinum-based catalysts. In the acidic solution the beginning voltage for the oxygen reduction process can be evaluated to be 0.97 V, which corresponds to 0.37 V as a minimum overvoltage for the ORR. The B_2_–SiNTs (7, 0) are suggested as an ORR catalyst in acidic environments.

## Abstract 2

Here, the potential of boron doped silicon nanotubes (7, 0) as ORR catalysts is examined.

## Graphical abstract

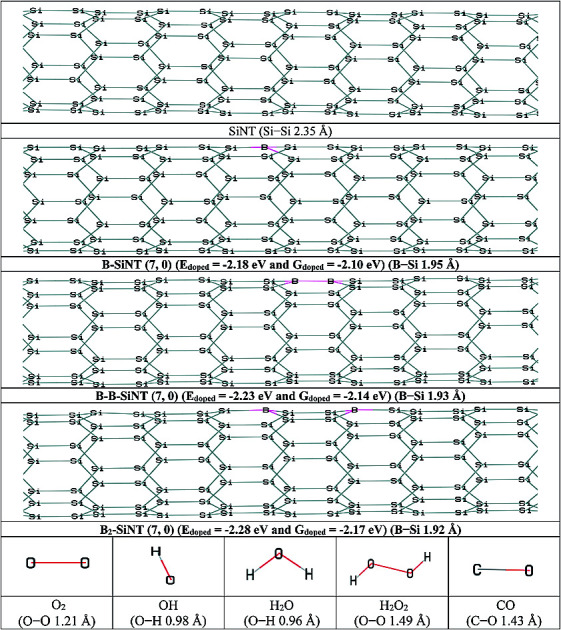

## 1. Introduction

Fuel cells as energy machines are important due to their low contamination and great efficiency. The ORR rate in electrodes of cells is slow, therefore the ORR can be evaluated as a significant reason to increase the full cell efficiency.^1–4^ Platinum-compounds have been used as catalysis in the ORR but platinum-compounds have low ability to endure CO.^5–9^

The potential of various compounds was investigated to find and propose effective catalysts for the ORR. Nanostructures and doped nanostructures with high ability for CO endurance can be used as suitable replacements for platinum-compounds.^10–15^ The B-nanostructures are acceptable catalysts for the ORR in alkaline conditions and mechanisms of action of B-doped nanostructures in acidic position are not clear.^16–23^

The nanostructures due to their electrical conductivity and thermal conductivity can be used to product the transistors and non-volatile memory devices.^24–28^ The electrical conductivity of doped carbon/silicon nanotubes indicated that the adoption of carbon/silicon nanotubes (with various atoms such as B, N, O and some metals) increased their electrical conductivity, significantly. These findings improved the application of carbon/silicon nanotubes in nano-electronic devices and novel catalyst to ORR.^29–38^ Results demonstrated that the adoption of carbon/silicon nanotubes increased their electrical conductivity and enhanced the ORR efficiency.^39–48^

Wang *et al.*^49^ demonstrated that boron-doped graphene nanoribbons are suitable catalyst to ORR catalyst. Xiao *et al.*^50^ proved that the layered silicon–carbon nano sheets represented the high activity in ORR without CO poisoning. Xia and Zhang *et al.*^51,52^ investigated the mechanisms of ORR of fuel cells in acidic environment on graphene cathodes. Stevenson *et al.*^53^ proved in ORR the O_2_ in a 2-electron path is reduced to form OOH on carbon nanotubes. Hu and Xiong *et al.*^54,55^ confirmed that nitrogen and boron-doped nanostructures as ORR catalysts have low price, great durability and excellent potential. Zhao and Wei *et al.*^56,57^ confirmed the doping of carbon nanotubes have vital roles on performance of ORR. Ferrighi *et al.*^58^ demonstrated that boron atoms of nano-sheets increase the reactions of oxygen with graphene.

In current study, ORR on B-doped silicon nanotube (7, 0) as acceptable catalysts is examined to find possible mechanisms to ORR on B_2_–SiNT (7, 0) and to suggest high activity nano-catalysts to ORR.

## 2. Computational details

In this study the silicon nanotube (length and diameter are 1 and 0.475 nm) is modeled and their open elements are saturated with hydrogen atoms to elude border effects. The geometries of nanotubes and studied molecules (such as OOH, OH, H_2_O and CO) are optimized by M06-2X/6-311G+ (2d, 2p) in GAMESS package.^59–72^ The consistent field is investigated by 10^−6^ Hartree as convergence value. Vibrational frequencies of nanotubes and molecules by M06-2X/6-311G+ (2d, 2p) are calculated.

In the density functional, M06 functionals are extremely parameterized proximate exchange functionals theory and they are supported on generalized gradient approximation (meta-GGA). These functionals are used for traditional quantum chemistry, solid-state physics calculations and thermodynamic values of reactions.^73–82^ M06-2X as the most accurate functional of Minnesota functional is a Global hybrid functional with 54% HF exchange and it is the ascendancy constructor within the 06 functionals for thermochemistry, kinetics and various chemical interactions.^73–82^ The M06-2X functional as hybrid meta exchange–correlation functionals present 32 empirically improved factors within the exchange–correlation functional.^83–87^

The energy and Gibbs free energy (*G* = *E*_0_ + ZPE + Δ*H* + *RT* − *TS*) values of nanotubes are calculated. The *E*_0_ and ZPE are electronic energy and zero-point energy and *T* is 298.15 K.^59–72^ Adoption energy (*E*_doped_) and Gibbs free energy adoption (*G*_doped_) of B atoms in SiNT (7, 0) are calculated:

1 *E*_doped_ = *E*(B–SiNT) − *E*(SiNT) − *E*(B)

2 *G*_doped_ = *G*(B–SiNT) − *G*(SiNT) − *G*(B)

3 *E*_doped_ = *E*(B_2_–SiNT) − *E*(SiNT) − 2*E*(B)

4 *G*_doped_ = *G*(B_2_–SiNT) − *G*(SiNT) − 2*G*(B)

*E*(B–SiNT (7, 0)) and *E*(B_2_–SiNT (7, 0)) are energies of B–SiNT (7, 0) and B_2_–SiNT (7, 0).

Energy adsorption (Δ*E*_ad_) and Gibbs free energy adsorption (Δ*G*_ad_) of molecules (such as OOH, OH, H_2_O and CO) on surfaces of studied nanotubes (SiNT, B–SiNT, B–B–SiNT and B_2_–SiNT) are calculated:

5 Δ*E*_ad_ = *E*(molecule–nanotube) − *E*(nanotube) − *E*(molecule)

6 Δ*G*_ad_ = *G*(molecule–nanotube) − *G*(nanotube) − *G*(molecule)

*E*(molecule–nanotube) and *G*(molecule–nanotube) are *E* and *G* of molecules–nanotubes. *G*(nanotube) and *G*(molecule) are *G* of nanotubes and molecules. *E*(nanotube) and *G*(molecule) are energy and *G* of nanotubes and molecules. Natural bond orbital charges (*q*) and gap energy (*E*_HLG_) complexes are calculated^59–72^ and transition state, reaction energy (Δ*E*_r_) and activation barrier energy (Δ*E*_a_) are examined by LST/QST method and M06-2X.^64^

Activation energy (Δ*E*_a_ = *E*_TS_ − *E*_IS_) is difference of energy between transition (*E*_TS_) and initial (*E*_IS_) studied complexes. In this study, the reaction energy (Δ*E*_a_ = *E*_FS_ − *E*_IS_) is difference of energy between final (*E*_FS_) and initial (*E*_IS_) studied complexes.^88–95^ The Δ*G* of ORR on B_2_–SiNT (7, 0) in according to standard hydrogen electrode was evaluated through Δ*G* = Δ*E* + ΔZPE − *T*Δ*S* + Δ*G*_U_ + Δ*G*_pH_ (obtained data were reported in Fig. 5).^96–104^ Δ*G*_pH_ (Δ*G*_pH_ = *kT* ln 10 × pH) is modification of proton Gibbs free energy and Δ*G*_U_ = − *neU*. The *n*, *e* and *U* are electrons, first charge and electrode potential. The *U* is requested potential and overvoltage is *η* = *U*_0_ − *U*.^49–58^ Conductor like screening method is used to estimate water environment (dielectric constant is 78.54).^59–72^

## 3. Results and discussion

### 3.1. Molecule adsorptions on nanotube

**Fig. 1:**
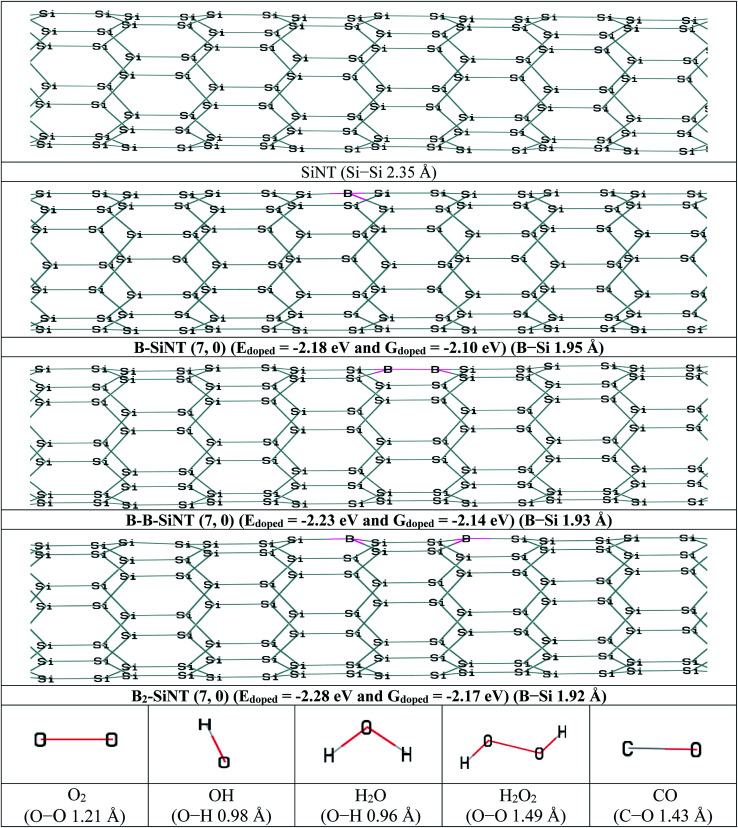
The initial structures of SiNT, B–SiNT, B–B–SiNT and B_2_–SiNT and O_2_, OH, H_2_O, H_2_O_2_ and CO molecules.

In this section, the B adoption of SiNT (7, 0) were investigated and then interactions of B–SiNT (7, 0) structures with O_2_, OOH, OH, H_2_O and CO molecules were investigated. The one Si atom of the SiNT (7, 0) was replaced with one B atom and the B–SiNT (7, 0) was produced (Fig. 1). Also the two Si atoms of the SiNT (7, 0) in two difference positions were replaced with two B atoms and B–B–SiNT (7, 0) and B_2_–SiNT (7, 0) structures were produced (Fig. 1). Structures of SiNT, B–SiNT, B–B–SiNT, B_2_–SiNT, O_2_, OH, H_2_O, H_2_O_2_ and CO are presented in Fig. 1. The adoption energy (*E*_doped_), adoption free Gibbs energy (*G*_doped_) and bond lengths of B–Si of B–SiNT (7, 0), B–B–SiNT (7, 0) and B_2_–SiNT (7, 0) were reported in Fig. 1. In the B–SiNT (7, 0) the B atom was connected with three neighboring silicon atoms and the *E*_doped_ and *G*_doped_ were −2.18 and −2.10 eV and average of bonds of B–Si in B–SiNT (7, 0) is 1.95 Å. In the B–B–SiNT (7, 0) the B atoms are connected with four neighboring silicon atoms and the *E*_doped_ and *G*_doped_ are −2.23 and −2.14 eV and average of bonds of B–Si in B–B–SiNT (7, 0) is 1.93 Å. In the B_2_–SiNT (7, 0) the B atoms are connected with six neighboring silicon atoms and the *E*_doped_ and *G*_doped_ values are −2.28 and −2.17 eV and average of bonds of B–Si in B_2_–SiNT (7, 0) are 1.92 Å.

The *q* and *E*_HLG_ of SiNT (7, 0), B–SiNT (7, 0), B–B–SiNT (7, 0) and B_2_–SiNT (7, 0) are stated in Table 1. *E*_HLG_ of SiNT (7, 0), B–SiNT (7, 0), B–B–SiNT (7, 0) and B_2_–SiNT (7, 0) are 1.84, 1.75, 1.68 and 1.64 eV. The *q* of B–SiNT (7, 0), B–B–SiNT (7, 0) and B_2_–SiNT (7, 0) are 0.58, 0.69 and 0.73|*e*|. The |*E*_doped_|, |*G*_doped_| and q values of B_2_–SiNT (7, 0) are higher than B–SiNT (7, 0) and B–B–SiNT (7, 0). Results showed that the *E*_HLG_ value of B_2_–SiNT (7, 0) is lower than corresponding values on surfaces of B–SiNT (7, 0) and B–B–SiNT (7, 0). Therefore, it can be concluded that the B_2_–SiNT (7, 0) is the most stable than B–SiNT (7, 0) and B–B–SiNT (7, 0) from thermodynamic view point. The B atoms in B–SiNT (7, 0), B–B–SiNT (7, 0) and B_2_–SiNT (7, 0) structures are advantageous to adsorption of O_2_ molecule and these B atoms are active positions of B–SiNT (7, 0), B–B–SiNT (7, 0) and B_2_–SiNT (7, 0) as catalyst for ORR. Therefore, B atoms are essential location to adsorption of O_2_ molecule and B atoms can be considered as initiator of first ORR step.

**Table 1:** Charge transfer (*q*) (in |*e*|) and HOMO–LUMO band gap (*E*_HLG_) (in eV) of studied complexes

Complex	*q*	*E*_HLG_	Complex	*q*	*E*_HLG_	Complex	*q*	*E*_HLG_	Complex	*q*	*E*_HLG_
*SiNT*	—-	1.84	*B*–*SiNT*	0.58	1.75	*B–B–SiNT*	0.69	1.68	*B_2_–SiNT*	0.73	1.64
*2a*	0.36	1.69	*2b*	0.47	1.61	*2c*	0.59	1.52	*2d*	0.64	1.48
*2e*	0.29	1.75	*2f*	0.39	1.68	*2g*	0.51	1.59	*2h*	0.58	1.55
*2i*	0.32	1.72	*2j*	0.43	1.64	*2k*	0.54	1.56	*2l*	0.61	1.52
*2m*	0.49	1.35	*2n*	0.62	1.23	*2o*	0.73	1.17	*2p*	0.82	1.14
*3a*	0.41	1.54	*3b*	0.51	1.45	*3c*	0.65	1.38	*3d*	0.73	1.25
*3e*	0.59	1.14	*3f*	0.81	1.05	*3g*	0.84	0.99	*3h*	0.91	0.95
*3m*	0.11	1.80	*3n*	0.14	1.70	*3o*	0.19	1.62	*3p*	0.21	1.60
*3q*	0.08	1.82	*3r*	0.12	1.72	*3s*	0.15	1.64	*3t*	0.18	1.63

**Fig. 2:**
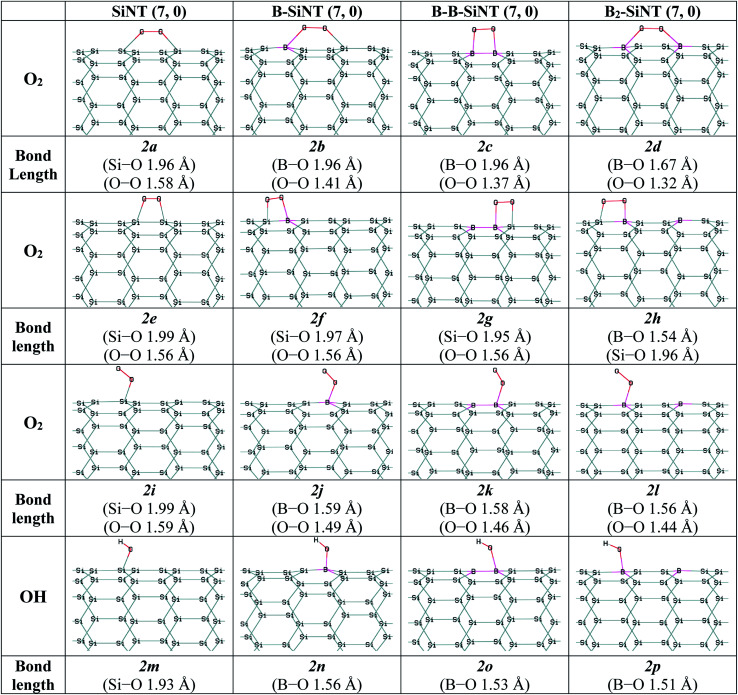
Complexes of the SiNT (7, 0), B–SiNT (7, 0), B–B–SiNT (7, 0) and B_2_–SiNT (7, 0) with O_2_ and OH molecules.

Therefore, O_2_ adsorption on SiNT (7, 0), B–SiNT (7, 0), B–B–SiNT (7, 0) and B_2_–SiNT (7, 0) were investigated. The possible positions of SiNT (7, 0), B–SiNT (7, 0), B–B–SiNT (7, 0) and B_2_–SiNT (7, 0) to O_2_ adsorption including top position B atom and bridge positions of B–Si, B–B and Si–Si bonds were examined in Fig. 2. The B–O, O–O and Si–O in SiNT (7, 0), B–SiNT (7, 0), B–B–SiNT (7, 0) and B_2_–SiNT (7, 0) with O_2_ are presented in Fig. 2 (*2a–2l* structures). The Δ*E*_ad_ and Δ*G*_ad_ of O_2_ on SiNT (7, 0), B–SiNT (7, 0), B–B–SiNT (7, 0) and B_2_–SiNT (7, 0) are displayed in Table 2. |Δ*G*_ad_| of O_2_ on B–SiNT, B–B–SiNT and B_2_–SiNT are greater than SiNT (7, 0). The |Δ*E*_ad_| and |Δ*G*_ad_| of O_2_ on B_2_–SiNT (7, 0) are greater than B–SiNT (7, 0), B–B–SiNT (7, 0). The bridge position of B–B in B_2_–SiNT (7, 0) is stable than top position B in B_2_–SiNT (7, 0) to O_2_ adsorption.

**Table 2:** The Δ*E*_ad_ (in eV) and Δ*G*_ad_ (in eV) values of studied complexes

Complex	Δ*E*_ad_	Δ*G*_ad_	Complex	Δ*E*_ad_	Δ*G*_ad_	Complex	Δ*E*_ad_	Δ*G*_ad_	Complex	Δ*E*_ad_	Δ*G*_ad_
*2a*	−0.41	−0.36	*2b*	−0.61	−0.54	*2c*	−0.84	−0.79	*2d*	−0.88	−0.81
*2e*	−0.39	−0.33	*2f*	−0.42	−0.38	*2g*	−0.55	−0.51	*2h*	−0.61	−0.55
*2i*	−0.27	−0.24	*2j*	−0.64	−0.61	*2k*	−0.69	−0.64	*2l*	−0.73	−0.68
*2m*	−2.07	−1.97	*2n*	−2.23	−2.15	*2o*	−2.31	−2.21	*2p*	−2.39	−2.28
*3a*	−0.87	−0.81	*3b*	−1.06	−0.99	*3c*	−1.11	−1.03	*3d*	−1.17	−1.12
*3e*	−3.11	−2.97	*3f*	−3.59	−3.45	*3g*	−3.67	−3.56	*3h*	−3.79	−3.67
*3i*	−2.24	−2.13	*3j*	−2.25	−2.15	*3k*	−2.27	−2.17	*3l*	−2.31	−2.21
*3m*	−0.15	−0.10	*3n*	−0.18	−0.13	*3o*	−0.20	−0.15	*3p*	−0.24	−0.19
*3q*	−0.09	−0.05	*3r*	−0.10	−0.07	*3s*	−0.11	−0.08	*3t*	−0.14	−0.12

Wang, Xiao and Xia *et al.*^49–51^ calculated the O_2_ adsorption on surfaces of boron-doped graphene nanoribbon, silicon–carbon nano sheets and B and N doped-graphene by theoretical methods, respectively (results reported in Table 4). The Δ*E*_ad_ of O_2_ on B-doped graphene, silicon–carbon nano-sheets and N-doped graphene are −0.62, −0.53 and −0.60 eV. Therefore Δ*E*_ad_ value (−0.61 eV) of O_2_ on B_2_–SiNT (7, 0) in present study is similar to corresponding values of O_2_ on various nanostructures were calculated in previous theoretical works.^49–51^

**Table 3:** The Δ*E*_a_ and Δ*E*_r_ for ORR on B_2_–SiNT (7, 0)

Path	Studied reaction steps	Δ*E*_a_ (eV)	Δ*E*_r_ (eV)
1	O_2_ + B_2_–SiNT (7, 0) → B_2_–SiNT (7, 0)–*O_2_	—	−0.68
1	B_2_–SiNT (7, 0)–*O_2_ + H^+^ + e^−^ → B_2_–SiNT (7, 0)–*OOH	0.00	−1.07
1	B_2_–SiNT (7, 0)–*OOH + H^+^ + e^−^ → B_2_–SiNT (7, 0)–*O + H_2_O	0.18	−2.75
1	B_2_–SiNT (7, 0)–*O + H^+^ + e^−^ → B_2_–SiNT (7, 0)–*OH	0.37	−1.57
1	B_2_–SiNT (7, 0)–*OH + H^+^ + e^−^ → B_2_–SiNT (7, 0)* + H_2_O	0.07	−1.34
2	O_2_ + B_2_–SiNT (7, 0) → B_2_–SiNT (7, 0)–*O_2_	—	−0.68
2	B_2_–SiNT (7, 0)–*O_2_ + H^+^ + e^−^ → B_2_–SiNT (7, 0)–*OOH	0.00	−1.07
2	B_2_–SiNT (7, 0)–*OOH + H^+^ + e^−^ → *OH–B_2_–SiNT (7, 0)–*OH	0.24	−2.97
2	*OH–B_2_–SiNT (7, 0)–*OH + H^+^ + e^−^ → B_2_–SiNT (7, 0)–*OH + H_2_O	0.35	−1.20
2	B_2_–SiNT (7, 0)–*OH + H^+^ + e^−^ → B_2_–SiNT (7, 0)* + H_2_O	0.07	−1.34

**Table 4:** The Δ*E*_ad_ (in eV) values of O_2_, O, OH and OOH on B-doped graphene, silicon–carbon nano-sheets and N-doped graphene^65–67^ and B–SiNT (7, 0), B–B–SiNT (7, 0) and B_2_–SiNT (7, 0) in this study

Catalysts/species	B-doped graphene^49^	Si–C nano-sheet^50^	N-doped graphene^51^	B–SiNT (7, 0)	B–B–SiNT (7, 0)	B_2_–SiNT (7, 0)
O_2_	−0.62	−0.53	−0.60	−0.69	−0.84	−0.88
O	−3.74	−4.11	−3.55	−3.59	−3.67	−3.79
OH	−2.38	−2.87	−2.41	−2.23	−2.31	−2.39
OOH	−1.12	−1.18	−1.06	−1.06	−1.11	−1.17

The charge transfer (*q*) and HOMO–LUMO band gap (*E*_HLG_) of the complexes of SiNT (7, 0), B–SiNT (7, 0), B–B–SiNT (7, 0) and B_2_–SiNT (7, 0) with O_2_ molecule are displayed in Table 1. The *E*_HLG_ values of O_2_ adsorption on B–SiNT (7, 0), B–B–SiNT (7, 0) and B_2_–SiNT (7, 0) are lower than SiNT (7, 0). The *E*_HLG_ values of O_2_ adsorption on B_2_–SiNT (7, 0) are lower than B–SiNT (7, 0), B–B–SiNT (7, 0). The bridge position of B–B in B_2_–SiNT (7, 0) has higher *q* and lower *E*_HLG_ than top position B in B_2_–SiNT (7, 0) to O_2_ adsorption. Complex of B_2_–SiNT (7, 0) with O_2_ molecule (*2d* structure) is the most stable than other complexes of B–SiNT (7, 0) and B–B–SiNT (7, 0) with O_2_ molecule from thermodynamic view point. It can be concluded that O_2_ adsorbed on B_2_–SiNT (7, 0) in figure *2d* significantly and there are suitable interactions between the O_2_ molecule and B_2_–SiNT (7, 0) and the adsorption of O_2_ molecules on studied surfaces are chemical adsorption processes.

**Fig. 3:**
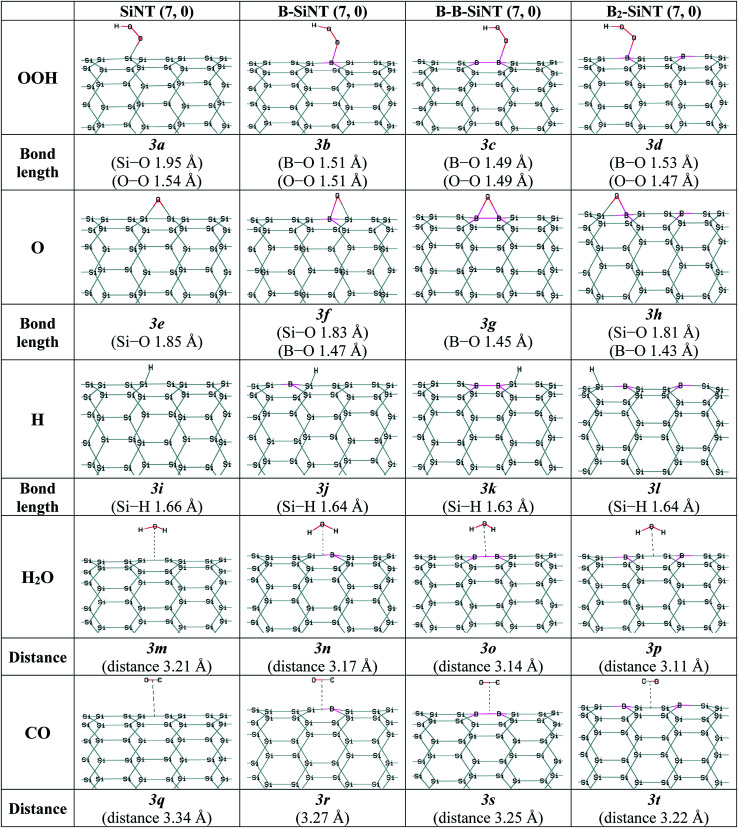
Complexes of the SiNT (7, 0), B–SiNT (7, 0), B–B–SiNT (7, 0) and B_2_–SiNT (7, 0) with O and H atoms, OOH, H_2_O and CO molecules.

In this study the interactions of important intermediates such as O, H, OOH, OH, H_2_O and CO molecules with SiNT (7, 0), B–SiNT (7, 0), B–B–SiNT (7, 0) and B_2_–SiNT (7, 0) surfaces in process of ORR were investigated (Fig. 2 and 3). The bonds of Si–O of SiNT (7, 0), B–SiNT (7, 0), B–B–SiNT (7, 0) and B_2_–SiNT (7, 0) with molecules are stated. *E*_HLG_, *q*, Δ*E*_ad_, Δ*G*_ad_ of molecules on SiNT (7, 0), B–SiNT (7, 0), B–B–SiNT (7, 0) and B_2_–SiNT (7, 0) are reported in Tables 1 and 2. OOH and OH intermediates can be adsorb on B site of B–SiNT (7, 0), B–B–SiNT (7, 0) and B_2_–SiNT (7, 0). The O intermediate has tendency to adsorb on B–Si and B–B bridge positions of B–SiNT (7, 0), B–B–SiNT (7, 0) and B_2_–SiNT (7, 0). It can be concluded that the complexes of B_2_–SiNT (7, 0) with O, H, OOH, OH and H_2_O molecules are stable than SiNT (7, 0), B–SiNT (7, 0) and B–B–SiNT (7, 0).

Wang, Xiao and Xia *et al.*^49–51^ calculated the O, OH and OOH adsorption on surfaces of boron-doped graphene nanoribbon, silicon–carbon nano sheets and B and N doped-graphene by theoretical methods, respectively (results reported in Table 4). Δ*E*_ad_ of O on B-doped graphene, silicon–carbon nano-sheets and N-doped graphene were −3.74, −4.11 and −3.55 eV. Δ*E*_ad_ of OH on boron-doped graphene, silicon–carbon nano-sheets and N-doped graphene were −2.38, −2.87 and −2.41 eV. Δ*E*_ad_ of OOH on B-doped graphene, silicon–carbon nano-sheets and N-doped graphene are −1.12, −1.18 and −1.06 eV. Δ*E*_ad_ values of O, OH and OOH (−1.17, −2.39 and −1.17 eV) on B_2_–SiNT (7, 0) in present study are similar to corresponding values of O, OH and OOH on various nanostructures were calculated in previous theoretical works.^49–51^

The H_2_O molecule favored to adsorb on above ring position of SiNT (7, 0), B–SiNT (7, 0), B–B–SiNT (7, 0) and B_2_–SiNT (7, 0) and the average of Δ*E*_ad_ and Δ*G*_ad_ values are −0.19 and −0.14 eV. The average of *q* and *E*_HLG_ values for adsorption of H_2_O molecule on SiNT (7, 0), B–SiNT (7, 0), B–B–SiNT (7, 0) and B_2_–SiNT (7, 0) surface is 0.16|*e*| and 1.68 eV. H_2_O molecule can be adsorbed on SiNT (7, 0), B–SiNT (7, 0), B–B–SiNT (7, 0) and B_2_–SiNT (7, 0) surfaces as physical adsorption processes.

In process of ORR the CO can occupy the positions of catalysts and the performance of ORR is reduced and efficiency of catalyst decreases sharply. Previous works showed that reactions between CO molecule and surface of platinum nano-catalyst was powerful (Δ*E*_ad_ is −1.90 eV) and CO poisoning was happen.^20,66^ The average of Δ*E*_ad_ and Δ*G*_ad_ of CO on SiNT, B–SiNT, B–B–SiNT and B_2_–SiNT surfaces are −0.11 and −0.08 eV. The average of *q* and *E*_HLG_ values for adsorption of CO on SiNT (7, 0), B–SiNT (7, 0), B–B–SiNT (7, 0) and B_2_–SiNT (7, 0) surface is 0.13|*e*| and 1.70 eV. The CO molecule can be adsorbed on SiNT (7, 0), B–SiNT (7, 0), B–B–SiNT (7, 0) and B_2_–SiNT (7, 0) surfaces as physical adsorption processes. It can be concluded that B_2_–SiNT (7, 0) as acceptable catalyst can be endurance to CO poisoning and it can solve the major problem of platinum nano-catalysts.

Wang, Xiao and Xia *et al.*^49–51^ calculated the H_2_O and CO adsorption on surfaces of boron-doped graphene nanoribbon, silicon–carbon nano sheets and B and N doped-graphene. The Δ*E*_ad_ of H_2_O on surfaces of B-doped graphene, silicon–carbon nano-sheets and N-doped graphene were −0.24, −0.18 and −0.08 eV. The Δ*E*_ad_ of CO on surfaces of B-doped graphene, silicon–carbon nano-sheets and N-doped graphene were −0.17, −0.07 and −0.12 eV. The Δ*E*_ad_ of H_2_O and CO (−0.24 and −0.14 eV) on B_2_–SiNT (7, 0) in present study are similar to corresponding values of H_2_O and CO on various nanostructures were calculated in previous theoretical works.^49–51^

### 3.2. B_2_–SiNT (7, 0) as catalyst to ORR

Nano-catalysts processed the chemical reactions through the ER and LH paths. The paths for ORR *via* B_2_–SiNT (7, 0) as acceptable catalyst through the LH and ER mechanisms were investigated. As start, O_2_ adsorption is investigated *via* O_2_ dissociation or hydrogenation of O_2_ to create B_2_–SiNT (7, 0)–*OOH. Firstly, the O_2_ dissociation process can be defined as B_2_–SiNT (7, 0)–*O_2_ → *O–B_2_–SiNT (7, 0)–*O. The dissociated O atoms were elected to link on B–Si position and activation barrier energy is 0.96 eV (figures *2a* (IS), *2b* (TS) and *2c* (FS)). Secondary, adsorbed O_2_ can interact *via* H atom to create B_2_–SiNT (7, 0)–*OOH as follow: B_2_–SiNT (7, 0)–*O_2_ + H^+^ + e^−^ → B_2_–SiNT (7, 0)–*OOH, this process has no any activation barrier energy.

The OOH adsorption on of B_2_–SiNT (7, 0) has higher Δ*E*_ad_ than O_2_*ca.* 0.29 eV and also O_2_ dissociation on surface of B_2_–SiNT (7, 0) has high activation barrier energy. H atom is added into Si in B_2_–SiNT (7, 0)–*OOH and H atom reacted *via* B_2_–SiNT (7, 0)–*OOH. Then the B_2_–SiNT (7, 0)–*OOH dissociated to *O–B_2_–SiNT (7, 0)–*OH (figures *2d* (IS), *2e* (TS) and *2f* (FS)), due to great activation barrier energy (1.31 eV) this process is impossible. The creation of B_2_–SiNT (7, 0)–*OOH in ORR on B_2_–SiNT (7, 0) is suitable than dissociation of O_2_ molecule.

The ORR is done through the B_2_–SiNT–*OOH intermediate as follows:

7 B_2_–SiNT–*OOH → B_2_–SiNT–*O + H_2_O

8 B_2_–SiNT–*OOH → *OH–B_2_–SiNT–*OH

9 B_2_–SiNT–*OOH → B_2_–SiNT + H_2_O_2_ →*OH–B_2_–SiNT–*OH

In path 1, B_2_–SiNT (7, 0)–*OOH intermediate was decreased to H_2_O molecule and B_2_–SiNT (7, 0)–*O (Δ*E*_a_ = 0.18 eV). In this process, O–O is fragmented and the first H_2_O molecule is created (figures *2g* (IS), *2h* (TS) and *2i* (FS)). Then two hydrogenation stages were done and B_2_–SiNT (7, 0)–*OH (figures *2j* (IS), *2k* (TS) and *2l* (FS)) and the second H_2_O molecule was created (figures *2m* (IS), *2n* (TS) and *2o* (FS)). The activation barrier energies of these two hydrogenation processes are 0.37 and 0.07 eV, respectively.

In path 2, H atom is linked to O and *OH–B_2_–SiNT (7, 0)–*OH is created and activation barrier energy is 0.24 eV (figures *2p* (IS), *2q* (TS) and *2r* (FS)). Then, *OH–B_2_–SiNT (7, 0)–*OH linked to H atom and the first H_2_O molecule is created and activation barrier energy of this stage is 0.35 eV (figures *2s* (IS), *2t* (TS) and *2u* (FS)). In the end stage of path 2, the B_2_–SiNT (7, 0)–*OH is hydrogenated and in this step the second H_2_O molecule is separated.

**Fig. 4:**
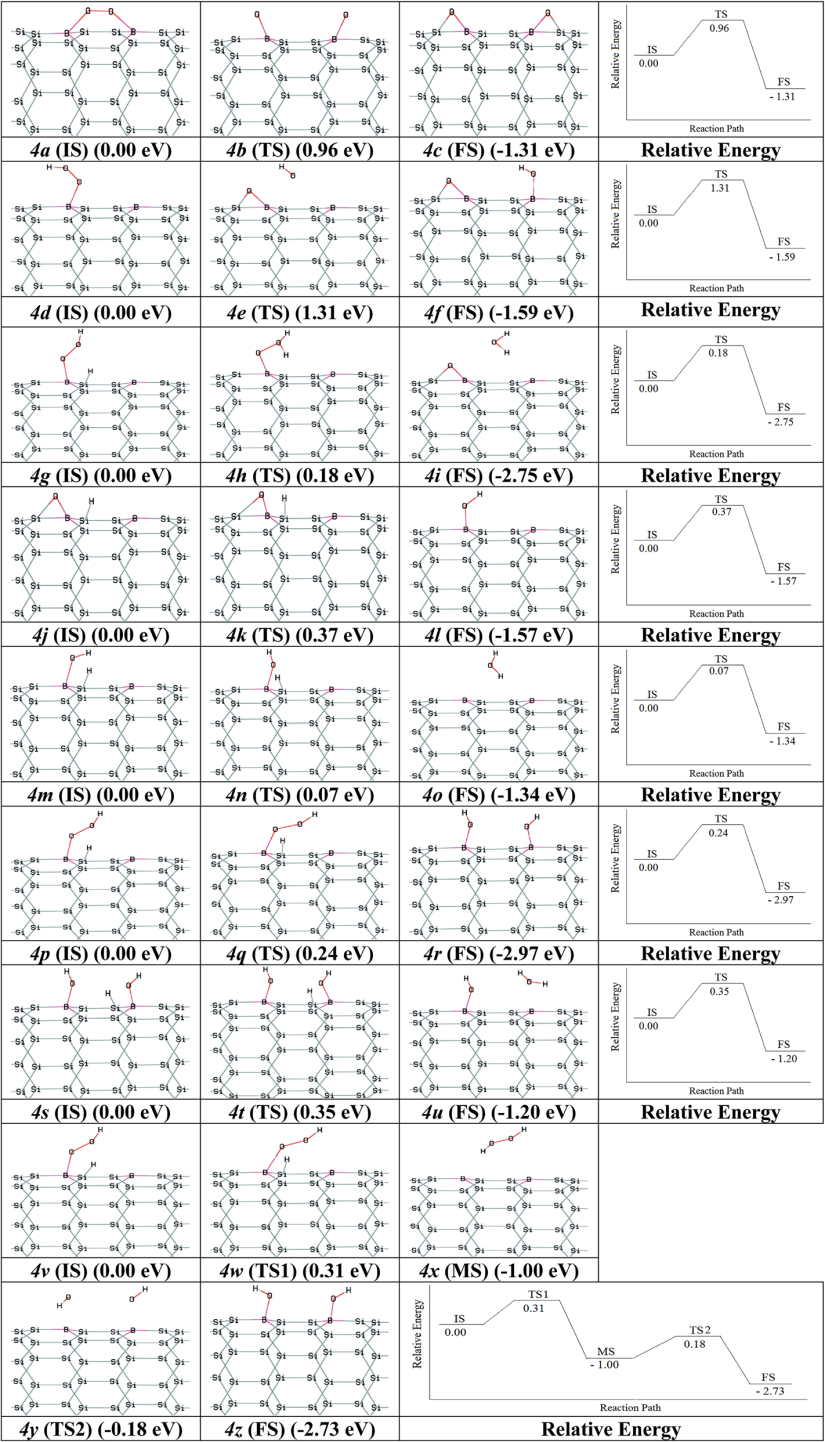
The intermediates of ORR and relative energies: (1) B_2_–SiNT (7, 0)–*O_2_ → *O–B_2_–SiNT (7, 0)–*O; (2) B_2_–SiNT (7, 0)–*OOH → *O–B_2_–SiNT (7, 0)–*OH; (3) B_2_–SiNT (7, 0)–*OOH → B_2_–SiNT (7, 0)–*O + H_2_O; (4) B_2_–SiNT (7, 0)–*O → B_2_–SiNT (7, 0)–*OH; (5) B_2_–SiNT (7, 0)–*OH → B_2_–SiNT (7, 0)–* + H_2_O; (6) B_2_–SiNT (7, 0)–*OOH → *OH–B_2_–SiNT (7, 0)–*OH; (7) *OH–B_2_–SiNT (7, 0)–*OH → *OH–B_2_–SiNT (7, 0) + H_2_O; (8) B_2_–SiNT (7, 0)–*OOH → B_2_–SiNT (7, 0) + H_2_O_2_ →*OH–B_2_–SiNT (7, 0)–*OH.

In the path 3, the B_2_–SiNT (7, 0)–*OOH is hydrogenated and the H_2_O_2_ molecule and B_2_–SiNT (7, 0) catalyst are created and activation barrier is 0.31 eV (figures *2v* (IS), *2w* (TS1), *2x* (MS), *2y* (TS2) and *2z* (FS)). The H_2_O_2_ molecule creation is a mediated state (MS) on surface of B_2_–SiNT (7, 0) and it cannot effect on potential of the B_2_–SiNT (7, 0), significantly. In next stage of path 3, separated H_2_O_2_ dissociated into *OH–B_2_–SiNT (7, 0)–*OH structure and therefore H_2_O_2_ dissociation has activation barrier energy about 0.82 eV. The ORR *via* path 3 continued *via* two hydrogenation stage as presented in path 1 in Fig. 4 (figures *4s* (IS), *4t* (TS) and *4u* (FS)) and path 2 in Fig. 4 (figures *4m* (IS), *4n* (TS) and *4o* (FS)).

The parameters of two acceptable paths about reduction of B_2_–SiNT (7, 0)–*OOH structure are stated in Table 3. In path 1, rate-determining stage (Δ*E*_a_ = 0.37 eV) on B_2_–SiNT (7, 0) surface is creation of B_2_–SiNT (7, 0)–*OH. In path 2, creation of B_2_–SiNT (7, 0)–*OH structure and H_2_O molecule is rate-determining stage (Δ*E*_a_ = 0.35 eV). In path 2, creation of *OH–B_2_–SiNT (7, 0)–*OH has higher Δ*E*_a_ than formation of B_2_–SiNT (7, 0)–*O + H_2_O in path 1 *ca.* 0.06 eV and so path 1 can be considered as optimal pathway to ORR.

**Fig. 5:**
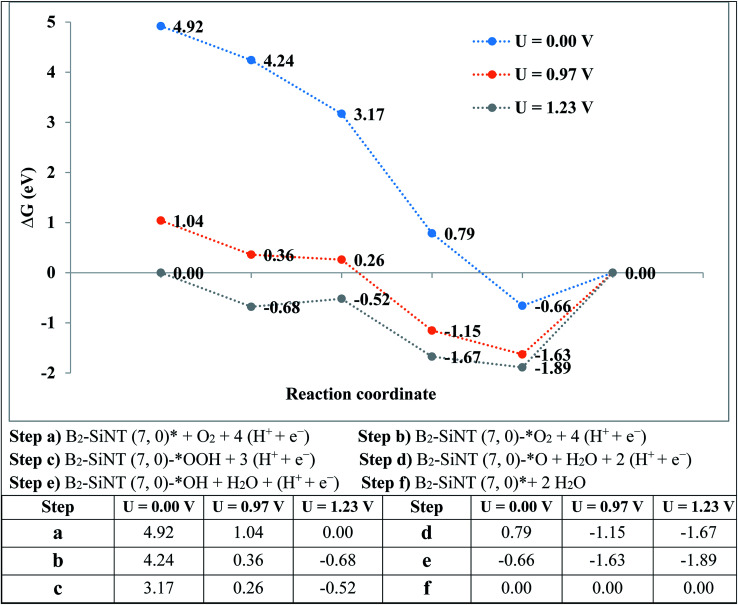
The *G* values for ORR on B_2_–SiNT (7, 0).

The over-potential of ORR on Pt-based compounds and graphene are 0.44 and 0.45 V.^49–59^ The experimental researchers investigated the onset-potential for the ORR performed on several catalysts^105–110^ and results are stated in Table 5. The *G* of ORR steps are stated in Fig. 5. The level of the final produce (B_2_–SiNT (7, 0)–* + 2H_2_O) is considered as reference step and ORR steps in *U* = 0 V is downhill. Reaction steps become downward that *U* is decreased to 0.97 V and beginning voltage for ORR is 0.97 V. The B_2_–SiNT (7, 0) is suggested as suitable ORR catalyst.

**Table 5:** The onset-potential (in eV) values for the ORR performed on several catalysts^105–110^

Catalyst	Onset potential	Catalyst	Onset potential	Catalyst	Onset potential
Pd/CNT^105^	0.764	Pd–Ni^107^	1.105	PdNi^109^	1.040
Pd/MWCNT^105^	1.014	PtCo/C^107^	0.836	Pd^109^	0.901
Pd–Ni(3 : 1)/C^106^	1.005	Pd–Fe/C^108^	0.865	Pd–Cu^110^	1.001
Pd_2_Co/C^106^	0.735	Pd/C^108^	0.920	Pt/C^110^	0.900

## 4. Conclusions

Performances of boron–silicon nanotube (7, 0) as novel catalyst to ORR are investigated. The ORR on surface of B_2_–SiNT can be continued through LH and ER mechanisms. The rate-determining stage (Δ*E*_a_ = 0.35 eV) for ORR on B_2_–SiNT (7, 0) surface is creation of B_2_–SiNT (7, 0)–*OH structure. The calculated beginning voltage to ORR on surface of the B_2_–SiNT (7, 0) is 0.37 V. In the acidic solution the beginning voltage to oxygen reduction process can be evaluated to 0.97 V. Results indicated that the B_2_–SiNT (7, 0) is suggested as catalyst to ORR with suitable efficiency.

## Conflicts of interest

There are no conflicts to declare.

## Supplementary Material
